# Death of Dr. Spurzheim

**Published:** 1833-02

**Authors:** 


### DEATH OF DR. SPURZHEIM.

John Gasper Spurzheim was born in December 1776, at the village of Longvitch, near the city of Treves, on the Moselle. His father was a farmer. He was designed by his friends for the profession of theology, and finished his education at the celebrated university of Treves. He subsequently became a tutor in a private family, and commenced his acquaintance with the well-known Dr. Gall, the founder of the craniological doctrine, as it was then called. In the year 1800, he attended, for the first time, the private course of lectures which Dr. Gall had been occasionally in the habit of giving, at his own residence, for four years past. Convinced that the principles advocated by Gall were founded in truth, and allured by the wide and uncultivated field of original research opened to his view, Spurzheim devoted himself particularly to anatomy and physiology, and, having completed his studies in 1804, became the associate and fellow-labourer of Dr. Gall. Previous to the commencement of this connexion, Gall had developed the principal points in the philosophy of his system, which may shortly be stated to be, firstly, that the moral qualities and intellectual faculties are innate; secondly, that their exercise, or their manifestation, depends upon the organization; thirdly, that the brain is the organ of all the propensities, mental emotions, and intellectual faculties; fourthly, that the brain consists of as many separate organs as there are propensities, feelings, and faculties, essentially differing from each other; and fifthly, that the form of the head, or cranium, represents, in the majority of cases, the form of the brain, and suggests varied means of ascertaining the primary qualities, and faculties, and the situation of their organs. Besides the development of these principles, Dr. Gall had pointed out the localities of the principal organs, and laid the foundation of his new anatomy of the brain.

Drs. Gall and Spurzheim, thus associated, unintermittingly pursued their inquiries; maturing their ideas, combating objections; multiplying observations, and examining the true structure of the brain. To this last department, it is understood, that Spurzheim's attention was at this time chiefly directed. In 1805, Dr. Gall was ordered to discontinue teaching his doctrine, or to quit Vienna: he chose the latter alternative, and, with his associate, set out on a journey through Europe. They visited the principal cities in Germany and the north of Europe, and arrived in Paris in 1807. In 1808, they presented a joint memoir on the Anatomy of the Brain to the Institute; and in their work they first described the true structure of the convolutions, and their connexion with the rest of the cerebral mass. Shortly after, they commenced and proceeded jointly in their great work, entitled "The Anatomy and Physiology of the Nervous System in general, and of the Brain in particular; with Observations upon the Possibility of ascertaining several Intellectual and Moral Dispositions of Man and Animals, by the Configurations of the Head," four vols. in folio, with an atlas of one hundred plates. During the publication of this magnificent work, some disagreement, it is alleged, occurred between the authors, and the work, which was not completed until 1819, was continued by Gall singly.

In 1814, Dr. Spurzheim visited England, and, by his lectures and writings, disseminated a knowledge of phrenology, as he now termed the science, and rendered its principles in some degree popular. A most violent attack was now made on the doctrine and its authors, by the late Dr. John Gordon, in the 49th number of the Edinburgh Review. "We look," says Dr. Gordon, "upon the whole doctrines taught by these modern peripatetics (Drs. Gall and Spurzheim,) anatomical, physiological, and physiognomical, as a piece of *thorough quackery* from beginning to end; they are a collection of new absurdities, without truth, connexion, or consistency, which nothing could have induced any man to present to the public, under pretence of instructing them, but absolute insanity, gross ignorance, or the most matchless assurance." To this criticism, Dr. Spurzheim published a calm and temperate reply. In 1817, he returned to Paris; and revisited England in 1825. Until his departure for America, he continued to give lectures in the principal cities of England, Ireland, and Scotland; and occasionally, during this period, passed his time at Paris.

About the time of his return to England, he married a French lady, but three or four years afterwards had the misfortune to lose his wife: she left no children.

During his residence in England, Dr. Spurzheim published the following works, some of which have passed through several editions: 1. The new Physiognomical System; 2. Phrenology, or the Doctrine of the Mind; 3. Philosophical Principles of Phrenology; 4. Outlines of Phrenology; 5. Elementary Principles of Education; 6. Examination of the Objections made in Great Britain against Phrenology; 7. Observations on Insanity; 8. Illustrations of Phrenology, in connexion with the study of Physiognomy; 9. A Catechism of Man: 10. The Anatomy of the Brain. Some of the views taken in these works by Dr. Spurzheim, differ from those advanced in the writings of Gall; and to the list of organs given by the latter, Dr. S. has added nine others. To these he has given the names Inhabitiveness, Conscientiousness, Hope, Marvellousness, Size, Weight or Resistance, Order, Eventuality, and Time.

A few months after, Dr. Spurzheim departed for the American Continent, and, having arrived at Boston, commenced a series of lectures. He had finished his course, with the exception of the concluding lecture, which he was prevented from finishing by severe indisposition, of which there had previously been some striking indication. His strength was evidently exhausted the last time he appeared in public; and when he announced his concluding lecture for a future evening, having, in the mean time been obliged to change his place of lecturing, and not having decided where he should assemble his hearers the next time, but designing to consult their wishes, he inquired of them "in what place shall we meet the next time?" a question which proved to be of sad and foreboding import. He did not live to meet those friends again, as he and they had fondly anticipated. The indisposition (continued fever) under which he was then suffering, gradually assumed a more severe character, and unhappily the state of his feelings produced a reluctance to call in medical aid, in the early stage of his illness; added to which was that inevitable anxiety of mind, which preys on the physical constitution in every one situated as he was, alone, and far remote from his native land. At length his physical powers, strong as they appeared to be, yielded to the disease, which perhaps operated also with augmented strength on a constitution of great susceptibility, and in a climate to which it was not habituated. This eminent man breathed his last on the 10th of November, 1832, in the fifty-eighth year of his age. The funeral took place on the 17th November, on which occasion, after the prayer, an eulogy was pronounced by Dr. Jollen, the German Professor of Harvard University, and an ode by the Rev. Mr. Pierpont was also sung.

The body was examined in the presence of many physicians, and the following appearances were found. The dura mater adhered to the cranium; pia mater was injected, but there was no effusion beneath it. Lungs healthy. The valves of the aorta were slightly thickened, and the caliber of the artery appeared dilated. The heart, a little larger than ordinary, was surrounded by a greater quantity of fat than usually exists in persons of Dr. Spurzheim's age. The ascending colon adhered to the abdominal parietes and the adjacent organs; but these adhesions did not appear to be of recent formation, although they were soft and yielding, and it did not seem probable that they had accelerated the fatal termination of the disease. The intestines were discoloured in various parts, but there was no alteration in the mucous membrane. Dr. Jackson, of Boston, who has published a detailed statement of Dr. Spurzheim's illness in an American Journal, remarks, at the conclusion of his account, that patients rarely die of idiopathic fever which is not complicated with some inflammatory disease, and that, therefore, the dissection was interesting in a pathological point of view, inasmuch as it furnishes one example of the existence of fever without inflammation.

